# School-based physical activity in relation to active travel – a cluster randomized controlled trial among adolescents enrolled in the school in motion study in Norway

**DOI:** 10.1186/s12966-023-01534-x

**Published:** 2023-11-21

**Authors:** Lena Malnes, Sveinung Berntsen, Elin Kolle, Andreas Ivarsson, Sindre M. Dyrstad, Geir K. Resaland, Runar Solberg, Tommy Haugen

**Affiliations:** 1 Department of Sport Science and Physical Education, University of Agder, Kristiansand, Norway; 2 Department Sports Medicine, Norwegian School of Sport Sciences, Oslo, Norway; 3 School of Health and Welfare, Halmstad University, Halmstad, Sweden; 4 Department of Education and Sport Science, University of Stavanger, Stavanger, Norway; 5 Faculty of Teacher Education and Sports, Western Norway University of Applied Sciences, Sogndal, Norway; 6 Centre for Epidemic Interventions Research, Norwegian Institute for Public Health, Oslo, Norway

**Keywords:** Green commuting, Active transport, Bicycling, Walking, Movement, Teenagers

## Abstract

**Background:**

Active travel and school settings are considered ideal for promoting physical activity. However, previous research suggests limited effect of school-based interventions on overall physical activity levels among adolescents. The relationship between physical activity in different domains remains inconclusive. In this study, we examined the effects of adding two weekly hours of school-based physical activity on active travel rates.

**Method:**

We analyzed data from 1370 pupils in the 9th-grade participating in the cluster RCT; the School In Motion (ScIM) project. Intervention schools (*n* = 19) implemented 120 min of class-scheduled physical activity and physical education, in addition to the normal 2 hours of weekly physical education in the control schools (*n* = 9), for 9 months. Active travel was defined as pupils who reported walking or cycling to school, while motorized travel was defined as pupils who commuted by bus or car, during the spring/summer half of the year (April–September), or autumn/winter (October–February). The participants were categorized based on their travel mode from pretest to posttest as; maintained active or motorized travel (“No change”), changing to active travel (motorized-active), or changing to motorized travel (active-motorized). Multilevel logistic regression was used to analyze the intervention effect on travel mode.

**Results:**

During the intervention period, most participants maintained their travel habits. In total, 91% of pupils maintained their travel mode to school. Only 6% of pupils switched to motorized travel and 3% switched to active travel, with small variations according to season and trip direction. The intervention did not seem to influence the likelihood of changing travel mode. The odds ratios for changing travel habits in spring/summer season were from active to motorized travel 1.19 [95%CI: 0.53–2.15] and changing from motorized to active travel 1.18 [0.30–2.62], compared to the “No change” group. These findings were consistent to and from school, and for the autumn/winter season.

**Conclusion:**

The extra school-based physical activity does not seem to affect rates of active travel among adolescents in the ScIM project.

**Trial registration:**

Clinicaltrials.gov ID nr: NCT03817047. Registered 01/25/2019′ retrospectively registered’.

**Supplementary Information:**

The online version contains supplementary material available at 10.1186/s12966-023-01534-x.

## Introduction

Low levels of physical activity are associated with increased risk of several chronic diseases [1]. Unfortunately, research have illustrated a high prevalence of low physical activity among European countries [2, 3]. In a study investigating objectively measured physical activity across 18 countries [2], the findings indicated that only 29% of adolescents were sufficiently active to meet the recommended level for physical activity, with 34% among Norwegian adolescents. To promote physical activity, interventions can be implemented in different domains such as during school hours, travel or leisure [4]. Schools have been perceived as an ideal context to promote physical activity due to their wide-reaching impact on the population, regardless of the adolescents’ diverse backgrounds [5, 6]. Research indicates that physical activity decreases with age [7, 8], and active travel may have a potential to alter the age-related decline in physical activity [7, 9].

There are various theories regarding how activity levels in one domain can influence physical activity in other domains. The ActivityStat hypothesis suggests that increased physical activity in one domain leads to a compensational effect by reducing physical activity in another domain [10]. Alternatively, the spillover refers to a cumulative effect which creates a positive spiral where increased activity leads to a further increase in other domains. Previous research has indicated a positive association between physical activity during travel and leisure time or sports [11–13]. Moreover, by becoming more physically active, one could improve physical fitness, which in turn is a determinant of physical activity [14]. Supporting the spillover effect, the Trans-Contextual Model [15] proposes that motivation and support achieved during physical education can transfer to motivation for activities in out-of-school domains, such as leisure or travel. Finally, the displacement hypothesis posits that increased sedentary time reduces physical activity [16], implying an inverse relationship between the two. In such, the displacement hypothesis could be used to argue that an increase in physical activity in one domain would equally increase physical activity overall.

While it may be assumed that increasing physical activity in one domain will lead to increased physical activity overall, the evidence supporting this notion has been inconsistent [10, 17]. In a recent review, Beck et al. [17] examined the displacement and compensation hypothesis, and synthesized findings from 77 studies investigating changes in physical activity among children and adolescents. Approximately half of the studies supported a compensation effect for increased physical activity. The findings also revealed that intervention studies, especially multi-component interventions and with longer intervention periods, were more likely to support compensation than observational studies. Additionally, compensation was more likely to occur in the school domain, followed by travel compared to organized sports [17]. While physical activity during school and travel are commonly treated as separate domains [4], it’s worth noting their close connection, as commuting to and from school is an integral part of many students’ daily routines and can be viewed as an extension of the school day. Considering the meta-analysis by Borde et al. [18] concluding that school-based interventions targeting adolescents had a minimal effect on overall physical activity, it is crucial to examine the effects of school interventions on other domains of physical activity in order to identify any compensatory effects.

The School in Motion project (ScIM) [19] was a school-based intervention aimed at increasing physical activity levels among Norwegian adolescents. The multi-component intervention involved adding an extra 120 minutes of class-scheduled physical activity during the school week in addition to the regular 2 hours of weekly physical education classes. To determine if class-scheduled physical activity during school influence active travel rates, we examined the effect of the ScIM intervention on travel mode to and from school, during the summer and winter season.

## Method

### Study design

The main aim, method, design and recruitment strategy in the ScIMs project have been presented elsewhere [19]. In brief, ScIM was a multicenter, three-arm cluster-randomized controlled trial conducted in the school year 2017–2018. The final sample consisted of 1370 adolescents in the 9th grade (14–15 years of age) from 28 schools (19 intervention schools), where each school was a cluster unit. The intervention was designed to increase physical activity by 120 minutes per week, in addition to the original classes of physical education (120–180 min/week). The intervention period lasted from September 2017 to June 2018, among all schools in the intervention group. Self-reported travel mode was collected at baseline in spring 2017 and approximately 12 months later in spring 2018.

Participating in the class-scheduled physical activity was mandatory for all pupils attending the intervention schools. The control schools received intervention material and funding the year after the data collection period was over. With respect to the data collection, an informed consent form was signed by parents, and the pupils could withdraw from data collection at any time. The ScIM project received approval from the Norwegian Centre for Research Data.

The ScIM project was commissioned and funded by the Norwegian Directorate for Education and Training (UDIR), although UDIR was not involved in its execution, data collection, analysis, or interpretation of findings.

### Sample

Of Norwegian public schools that met the inclusion criteria, we randomly invited schools to participate in ScIM. If a school declined the invitation, another school in the respective region was randomly selected and invited, until the target of 30 schools was included. Thereafter, 20 schools were randomly allocated to the intervention group and 10 to the control group. One control school withdrew from the study after randomization, and one intervention school withdrew after 12 weeks of performing the intervention, resulting in 28 schools in the final sample. We invited 2733 pupils whereas 2084 agreed to participate in the data collection (response rate 76%), and 1370 had valid pre-post data on travel habits in all four outcomes. Exclusion criteria for participation were that the schools had (1) less than 25 pupils in 9th grade, (2) any initiatives to increase mandatory physical activity during school hours, (3) participation in another comprehensive research project, and (4) private schools or (5) schools exclusively for pupils with special needs.

### Settings

The schools included in this study were located in various parts of Southern Norway, in the regions near each of the four test centers (Norwegian School of Sport Sciences, University of Stavanger, University of Agder and Western Norway University of Applied Sciences). The Statistics Norway [20] classifies municipalities based on their degree of centrality, ranging from 1 (most urban) to 6 (most rural), using the capital of Norway as the benchmark for the most urban score. Applying this classification, the included schools reside in areas defined as urban (*n* = 15), intermediate (*n* = 11), and rural (*n* = 2), with centrality index codes of 1–2, 3–4, and 5–6, respectively. It is important to note that Norway has a lower population density than most other European countries [21], which may affect how areas in Norway are perceived in an international perspective. Furthermore, the schools varied in size and consenting participants varied from 24 to 135 pupils in the 9th grade at each school.

### Intervention

The ScIM project had three study arms, with a two-arm intervention designed to increase physical activity by 120 minutes each school week. Both intervention arms involved extra 60 minutes of physical education and 60 minutes of physical activity, in addition to the standard classes of physical education (120–180 minutes per week). Since we aimed to examine school-based physical activity, rather than the specific attributes of the intervention components, we combined both intervention arms into a single group, hereby referred to as the intervention group. Detailed information about the intervention components for the two interventions, teachers training, and material used, has been presented elsewhere [19, 22].

During the intervention period, a teacher reported intervention adherence every week at each school through a website designed at the Norwegian School of Sport Sciences. The intervention schools completed on average 80% of the intended dose of physical activity through the intervention period. The CONSORT and the TIDieR checklist are attached as supplementary material.

### Measures

The participants reported travel mode to and from school, in the winter and summer seasons and answered the questionnaire on a computer. We used two items from the Health Behaviour of School-aged Children (HBSC) questionnaire [23]. Since both trip direction [24] and season [25, 26] can influence physical activity and active travel, we added seasonal and monthly specifications (summer and winter half of the year) to the questionnaire items and measured both to and from school. We have previously reported data on convergent validity for each item [27], comparing the HBSC questionnaire items to a five-day travel diary in the spring/summer season. In the present study, the participants answered the following question(s); “On a typical day in the spring/summer (April to September) is the main part of your journey to school made by …?” followed by a question capturing travel mode from school. The participants answered with the following options: by walking, by bicycle, by car/motorcycle/moped, by bus/trains/subway/ferry, or by other.

Data were categorized into two groups; those reporting cycling or walking were categorized as active travel, and car/motorcycle/moped or bus/trains/subway/ferry were categorized as motorized travel. Pupils that checked the box for “other” were not included in the analysis. Thereafter, we merged data from the pretest and posttest into one variable and categorized participants that 1) maintained active travel, 2) maintained motorized travel, 3) changed to active travel or 4) changed to motorized travel. Finally, we categorized the two groups that maintained their travel habits into one category, named “No change”.

We collected data on confounding variables which were ethnicity, age, gender, mother’s education, father’s education, and geographical region. We also collected data for descriptive purposes which were height, weight and accelerometer-measured physical activity.

### Statistical analysis

Baseline characteristics (age, height, weight, waist circumference, physical activity at baseline, gender, ethnicity, percent bicycling and walking) are presented as mean (SD) or median (IQR) or percent (N) as appropriate.

We use a categorical multivariate multilevel regression analysis, using a Bayesian estimator, to calculate the odds ratio of belonging to a group other than the control group. The multilevel setup, adjusting for potential clustering effects, was selected because the data is nested (individuals are nested in schools, schools are nested in regions). More specifically this is one solution to deal with the potential dependency that might be present in data that is collected from, for example, the same school. We ran the model with all four outcome variables within the same model.

Within the estimated model we adjusted for ethnicity, age, gender, mother’s education, father’s education, and geographical region. Within the multilevel analysis the cluster structure of the data (schools, regions) is specified within the model. In addition, we merged pre-post data and changes of travel habits was the outcome in our analysis. The “No change” group was used as reference level. Cases containing missing data were deleted for each model, meaning that the number of cases vary between models. The results are presented as median log-odds ratio with 95% credibility intervals (95% CI), given by the highest-density interval. The interpretation of the CI is that there is a 95% probability that the odds-ratio falls in this range. The interpretation of the Odds Ratio is that a value above 1 favors the intervention, and below 1 favors the control. All analyses were conducted in R where the models were fitted in Stan through the brms package using 4 chains, 2000 warm-up and 2000 sampling iterations. A description with the R-code used in the analysis is attached as supplemental material.

## Results

**Fig. 1:**
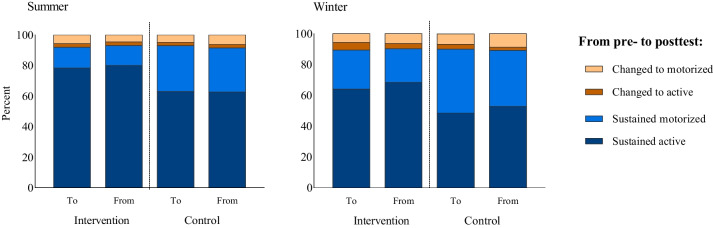
Travel patterns from pretest to posttest; to and from school divided by intervention- and control group, during summer- (left) and winter season (right)

Table 1 present descriptives of the participants in present study. In total, most participants engaged in active travel both to and from school, during spring/summer and autumn/winter season. Table 1 illustrates that the intervention group reported a higher rate of active travel (78%) commutes compared to the control group (63%) at baseline. Furthermore, 91% of pupils maintained their initial travel mode and 3% switched to active travel and 6% switched to motorized travel after 1 year. No significant differences were observed between the intervention and control group concerning changing travel mode (Fig. 1).

**Table 1:** Baseline characteristics of the sample stratified by intervention and control group. Data are presented as mean and standard deviation (SD), unless otherwise stated

	Control (***n*** = 494)	Intervention (***n*** = 876)
Girls, % (n)	51.6 (255)	52.2 (457)
International background, % (n)	9.5 (47)	7.6 (67)
Age (years)	14.0 (0.3)	13.9 (0.3)
Height (cm)	165.8 (7.6)	165.5 (7.9)
Weight (kg)	54.6 (10.5)	55.0 (10.3)
Waist circumference (cm) ^med^	67.2 (8.0)	67.0 (8.0)
Physical activity (minutes MVPA)	74.3 (28.4)	69.9 (26.3)
Active travelers in spring/summer		
To school		
by foot, % (n)	29 (141)	49 (425)
by bicycle, % (n)	39 (194)	36 (311)
From school		
by foot, % (n)	29 (144)	49 (433)
by bicycle, % (n)	40 (195)	35 (308)
Active travelers in autumn/winter		
To school		
by foot, % (n)	41 (202)	57 (502)
by bicycle, % (n)	14 (70)	12 (104)
From school		
by foot, % (n)	48 (236)	62 (547)
by bicycle, % (n)	14 (67)	12 (103)

*cm* centimeter, *kg* kilogram, *n* number, *%* percent, *MVPA* moderate to vigorous physical activity, med median (IQR). International background refers to participants born in another country than Norway

**Fig. 2:**
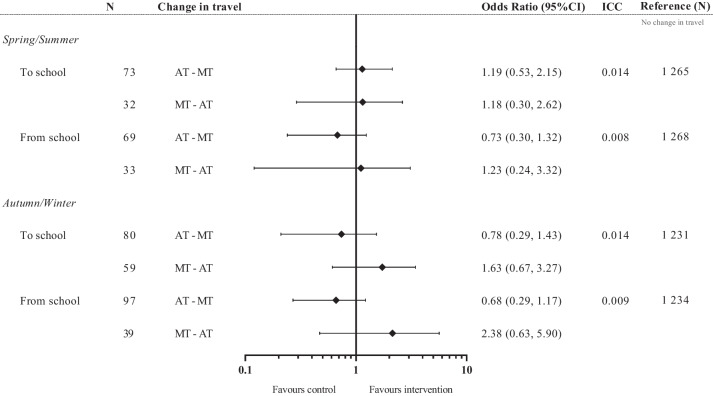
The likelihood (Odds ratio) of change in travel habits from active to motorized (AT-MT) or from motorized to active (MT-AT) compared to the participants who maintained their travel habits in the “No Change” group (AT-AT and MT-MT). Odds ratio (OR) below 1 favors control, and above 1 favors the intervention. “No change” is set as reference. N = number of participants. ICC = intraclass correlation

The analysis showed that the likelihood of changing travel modes was not different in the intervention group compared to the control group, as shown in Fig. 2. These findings remained consistent for both directions of travel (to and from school) and during winter seasons.

## Discussion

The aim of the present study was to determine the influence of extra 2 hours of physical activity during school on active travel rates among adolescents in 9th grade. During the ScIM intervention, most participants used active travel at baseline and maintained their travel habits throughout the intervention period. While there were differences between the intervention and the control group in terms of baseline active travel rates, the participants who changed their travel habits during the intervention period were similar in both groups (Fig. 1). This suggests that despite differences in baseline characteristics, the intervention did not influence changes in travel habits. The findings indicated that the extra physical activity and physical education did not negatively modify the adolescent’s transport-related activity outside of school.

There may be several possible explanations for our findings. Travel habits are repetitive behavior that can be an integrated part of a daily routine, especially travelling for commuting purposes. One might hypothesize that commuting can be more resistant to external influences. With respect to the ActivityStat hypothesis, we focused on commuting habits in the present study, so a potential compensation effect may have occurred when travelling for other purposes instead. Additionally, since the HBSC instrument captures the typical travel mode, we have investigated the main mode of travel. Smaller changes, for example reducing cycling from five to three days per week, may not be captured in this analysis. With respect to a spillover effect, the trans-contextual model suggests that if autonomous motivation towards an activity occurs in PE during school, the motivation can transfer to similar activities outside of school [15]. Therefore, the lack of spillover may be due to the lack of similarity between the activities in school and active travel, such as cycling. One may speculate if motivation towards cycling or walking would increase autonomous motivation with other activities or components. Moreover, health related fitness can be a strong predictor for future physical activity level [14], and health related fitness did increase in one of the two-arm intervention group in the ScIM project, as reported elsewhere [19]. Still, we observed minimal changes in travel habits during the intervention period. Although, in the other intervention model, a negative intervention effect on cardiorespiratory fitness was observed [19], which may moderate the potential effect of increased fitness. Overall, based on our findings on the relationship between school-based physical activity and active travel, the displacement hypothesis seems to be the theory most in accordance with our findings.

Findings from other studies have also indicated that physical activity, is not a determinant for active travel. The SPACE study [28] was a one-year school-based intervention that aimed to promote physical activity during recess, travel and leisure time among 11–14-year-old pupils. Although physical activity during recess increased in the intervention group [29], the mode of travel was maintained in both groups [28]. However, the SPACE intervention components were non-curricular, whereas the components in our intervention were mandatory for participants attending an intervention school. Also, in observational studies our findings have been supported as well [30, 31]. For instance, Foley et al. [30] investigated domain- specific physical activity in adults and found no association between changes in objective and subjective measured recreational physical activity and active travel to and from work. Although, sensitivity-analysis showed that women who increased cycling to work or had a higher BMI, were more likely to decrease recreational physical activity. In another study, Sahlqvist et al. [31] made a similar observation, although with an inverse perspective. They found that change in active travel does not influence changes in recreational physical activity. However, both Sahlqvist et al. [31] and Foley et al. [30] included adults, while the present study investigated a physical activity intervention among adolescents. Given that these studies all differed in with respect to population, study design and curriculum-based intervention components compared to the present study, more similar studies are needed to support our conclusions, preferably with a RCT design investigating class-scheduled physical activity.

Other aspects are important to consider when interpreting the results. For example, previous research indicated that long-lasting interventions over 1 year were more likely support the ActivityStat hypothesis [17], while our intervention lasted for 9 months. Moreover, in our study, the majority of adolescents used active travel modes at baseline, and there may be less potential for a further increase in active travel rates as a result of a potential spillover effect of increased school-based physical activity. Finally, previous research suggests that the school and transport domain were more likely to be compensated for than leisure or sport [17]. In this study, we focus on two domains: school and travel, and it is possible that any spillover or compensatory effects could have influenced leisure activities differently.

Overall, the results indicated that the additional curriculum physical activity was in addition to, and not instead of, active travel, which is promising as active travel among adolescence is a key contribution to the total physical activity level [32]. Therefore, our findings are relevant for schools planning to implement additional physical education or organized physical activity. Also, our study is relevant for other researchers seeking knowledge about determinants of active travel. Nevertheless, more studies with a longer intervention period are needed; and we recommend that future studies consider both travel mode, school- and leisure time activities in their analysis.

### Study strengths and limitations

The main strength of the present study is the cluster randomized controlled design, which is to date the best method to establish causal effects. The schools were included in the study before randomization, decreasing the risk of selection bias. Despite this, the groups were not equal at baseline, and we observed higher prevalence of active travel among adolescence attending an intervention school (Fig. 1). To address these inequalities, we adjusted for both cluster and baseline travel mode in the analysis.

Our main outcome was self-reported travel mode, where physical activity during active travel (cycling or walking) implies more activity than motorized travel. In relation to this, a study weakness is the lack of a measure of intensity of physical activity during transport. One may therefore speculate if the extra physical activity could have been compensated for by lesser intensity such as cycling or walking at a slower pace, or even fewer days per week as we only ask for “usual” travel mode in the questionnaire. In addition, the lack of data on travelled distance is another noteworthy limitation. Data on distance would enable us to calculate the amount active travel performed. Also, previous studies consistently illustrated that distance is a significant predictor of active travel [33], and the inclusion of data on distance to school could have provided insight in the feasibility and potential of a further increase in active travel, especially considering the high proportion of active travelers at baseline.

We strengthened the internal validity by establishing convergent validity of the questionnaire items [27], which in general seem uncommon in active travel research [34]. However, data from our previous publication on validation illustrated that motorized travel was underestimated [27], which may indicate a social desirability or recall bias. Moreover, while we had a large initial sample, there were less than 50 participants in some of the subgroups (Fig. 2) which lower the statistical power. Concerning external validity, the study had a high response rate of 76% among invited adolescents, which strengthens the representativeness of each school. On the other hand, 54 schools declined the invitation [19] which may limit representativeness of schools in Norway.

## Conclusion

The results of this study suggest that adding 2 hours of school-based physical activity per week did not have a significant impact on rates of active travel among adolescents in Norwegian public schools. This is an important finding given the potential for the ActivityStat hypothesis to undermine the effectiveness of physical activity interventions. Future research should continue to examine the potential impact of school-based physical activity interventions on travel habits, as well as leisure-physical activity, particularly in other contexts and among different populations.

### Supplementary Information

**Additional file 1.** Consort Checklist and Tidier-Checklist.

**Additional file 2.** R-code-multivariate-multilevel-logistic-regression.

## Data Availability

The data analysed during this study are not publicly available to protect the participants’ privacy, as they were insured in the consent form.

## Abbreviations

CI: Confidence intervals
Cm: Centimeters
C-RCT: Cluster-randomized controlled trial
HBSC: Health behavior of school aged children
IJBNPA: International journal of behavioral nutrition and physical activity
IQR: Interquartile range
Kg: Kilogram
MVPA: Moderate to vigorous intensity of physical activity
OR: Odds ratio
RCT: Randomised controlled trial
ScIM: School in motion project
SD: Standard deviation
Yrs: Years
