# Supplementary material for: School-based physical activity in relation to active travel – a cluster randomized controlled trial among adolescents enrolled in the school in motion study in Norway
Source: Int J Behav Nutr Phys Act. 2023 Nov 21;20:136. doi: 10.1186/s12966-023-01534-x (PMC10664674; doi:10.1186/s12966-023-01534-x)
Supplement: Supplementary file 2 — Additional file 2. R-code-multivariate-multilevel-logistic-regression. [file 12966_2023_1534_MOESM2_ESM.pdf]

R-code multivariate multilevel logistic regression

```r
# Load packages ---------------------------------------------------------------

library(haven)
library(dplyr)
library(brms)
library(tidyr)
library(tidybayes)


# Load data -------------------------------------------------------------------

# Reads the data from the working directory
data <- read_sav("data.sav")


# Data cleaning ---------------------------------------------------------------

data_clean <- data |>
  mutate(
    across(
      c(kjonn, Etn, Tsent),
      haven::as_factor
    ),
    across(
      starts_with("Endring"),
      haven::as_factor
    ),
    across(
      starts_with("3kat"),
```

```r
      \(x) relevel(x, ref = "No change")
    ),
    across(
      ses_fr:ses_mr,
      \(x) x - 3
    ),
    alder = scale(alder),
    Int_arm_2r = factor(
      Int_arm_2r,
      levels = c(2,1),
      labels = c("Kontroll", "Intervensjon")
    )
  ) |>
  drop_na(
    kjonn,
    alder,
    Etn,
    ses_fr,
    ses_mr,
    Int_arm_2r,
    Tsent,
    starts_with("Endring")
  )


# Create and run the model -------------------------------------------------

model_Endring <- brm(
  bf(
    mvbind(
      Endring_s7_3kat,
      Endring_s8_3kat,
      Endring_s9_3kat,
      Endring_s10_3kat
    ) ~ 1 +
      kjonn + alder + Etn + ses_fr + ses_mr + Int_arm_2r + Tsent + (1|p|Idsk)
  ),
  family = categorical(),
  data = data_clean,
  iter = 4000,
  chains = 4,
```

```r
  cores = 4,
  prior = prior(normal(0,2.5), class = "b"),
  control = list(adapt_delta = .95),
  backend = "cmdstanr"
)


# Extract fixed effects parameters ----------------------------------------

model_Endring %>%
  gather_draws(`b.*`, regex = TRUE) |>
  mutate(.value = exp(.value)) |>
  median_hdci() |>
  separate(
    .variable,
    into = c("b", "mu", "Endring", "var"),
    sep = "_"
  ) |>
  arrange(Endring) |>
  select(mu:.upper)


# Extract random effects parameters ---------------------------------------

model_Endring |>
  gather_draws(`sd.*`, regex = TRUE) |>
  median_hdci() |>
  separate(
    .variable,
    into = c("sd", "ldsk", "empty", "mu", "Endring", "var"),
    sep = "_"
  ) |>
  select(mu, Endring, .value:.upper)
```
